# Natural Product-Derived Drugs: Structural Insights into Their Biological Mechanisms

**DOI:** 10.3390/biom15091303

**Published:** 2025-09-10

**Authors:** Yujeong Choi, Younghyun Kim, Hye Joon Boo, Danbi Yoon, Jeong Seok Cha, Jiho Yoo

**Affiliations:** 1 College of Pharmacy, Chung-Ang University, Seoul 06974, Republic of Korea; 2 Research Institute of Pharmacy, Chung-Ang University, Seoul 06974, Republic of Korea

**Keywords:** natural products, structural biology, molecular mechanisms, X-ray crystallography, cryo-EM, protein-drug interactions, drug discovery

## Abstract

Natural product-derived drugs represent a cornerstone of modern pharmacotherapy, with many serving as essential therapeutic agents across diverse medical conditions. Recent advances in structural biology have provided unprecedented insights into the molecular mechanisms underlying their biological activities. This review presents a comprehensive structural analysis of five representative natural product-derived drugs: digoxin, simvastatin, morphine, paclitaxel, and penicillin. Through an examination of high-resolution crystal structures and cryo-electron microscopy (cryo-EM) data, we elucidate how these compounds interact with their respective protein targets and modulate biological functions. The structural data reveal diverse binding mechanisms—ranging from competitive inhibition and covalent modification to allosteric modulation via conformational selection and induced fit—demonstrating how natural products achieve their therapeutic effects through precise molecular recognition. These structural insights provide a molecular foundation for understanding natural product pharmacology and offer valuable guidance for structure-based drug design approaches in developing next-generation therapeutics.

## 1. Introduction

Naturalproducts have served as invaluable sources of therapeutic compounds throughout human history, providing the foundation for many of today’s essential medications [1,2,3]. These structurally diverse molecules, evolved through millions of years of natural selection, possess unique chemical scaffolds that often exhibit remarkable selectivity and potency against biological targets. The reported impact of natural products on drug discovery varies depending on the analysis criteria. Estimates suggest that between one-third and up to 65% of approved small-molecule drugs in the last few decades are derived from natural products [1].

This remarkable contribution spans virtually every therapeutic area. Aspirin, derived from willow bark (*Salix species*), revolutionized pain management and cardiovascular protection. Penicillin from *Penicillium* molds transformed infectious disease treatment. Paclitaxel from the Pacific yew (*Taxus brevifolia*) became a cornerstone of cancer chemotherapy. Morphine from the opium poppy (*Papaver somniferum*) remains the gold standard for severe pain relief. Recent analyses by Newman and Cragg indicate that between 1981 and 2019, natural products, their derivatives, and synthetic drugs designed based on natural product pharmacophores accounted for approximately 65% of small-molecule drugs approved by the U.S. Food and Drug Administration (FDA). This percentage has remained remarkably consistent across decades [1]. Furthermore, natural products serve as invaluable lead compounds for drug discovery, providing unique chemical scaffolds that have inspired the development of numerous synthetic analogs with improved therapeutic profiles [4].

The molecular mechanisms by which natural products exert their biological effects have long been a subject of intense scientific investigation. However, it is the advent of high-resolution structural biology techniques that has truly revolutionized our understanding of natural product-protein interactions [5]. X-ray crystallography, nuclear magnetic resonance (NMR) spectroscopy, and more recently, cryo-electron microscopy (cryo-EM) have enabled researchers to visualize these interactions at atomic resolution. These approaches reveal the precise molecular details that govern biological activity [5].

Our primary focus is structural–mechanistic insight; clinical applications are referenced to illustrate translation rather than to provide an exhaustive therapeutic review. This review focuses on five representative natural product-derived drugs that exemplify different therapeutic categories and molecular mechanisms: digoxin (cardiovascular), simvastatin (hyperlipidemia), morphine (analgesic), paclitaxel (anticancer), and penicillin (antibiotic). Each of these compounds has been extensively studied through structural biology approaches, providing detailed insights into their mechanisms of action. By examining the structural basis of their biological activities, we aim to illustrate how natural products achieve their therapeutic effects through precise molecular recognition. We also demonstrate the value of structural information in understanding drug-target interactions. The availability of high-resolution structural data for these natural product-protein complexes has opened new avenues for structure-based drug design and optimization [6].

Distinct from prior reviews, we provide a mechanism-centric, cross-target comparison that unifies competitive inhibition, covalent acylation, conformational selection, and induced fit across five canonical natural products, and we explicitly integrate recent cryo-EM, molecular dynamics (MD), and machine learning (ML) insights to link static structures to dynamics and function.

## 2. Structural Analysis of Natural Product-Protein Complexes

### 2.1. Digoxin and Na^+^/K^+^-ATPase: Mechanisms of Ion Transport Inhibition

**Figure 1:**
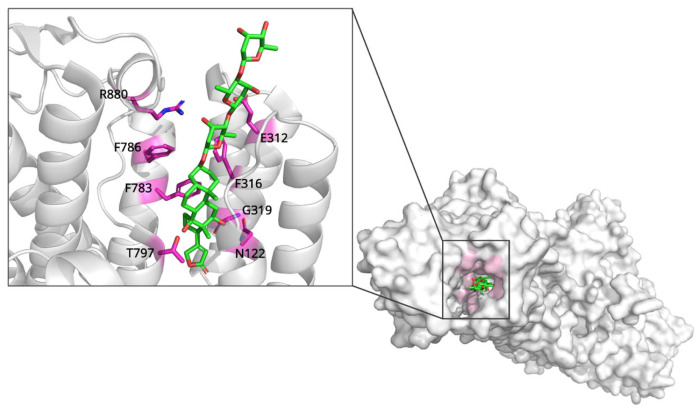
The complex structure of Na^+^/K^+^-ATPase with digoxin. The preformed cavity within Na^+^/K^+^-ATPase is represented at extracellular domain. Key residues that interact with digoxin in preformed cavity are labeled at enlarged view. Figure was created by PyMOL program with PDB ID: 7DDH.

Digoxin, a cardiac glycoside from *Digitalis lanata*, is one of the oldest heart failure drugs; its mechanism was clarified only recently by structural studies [7]. The target of digoxin is the Na^+^/K^+^-ATPase, a membrane-bound enzyme responsible for maintaining cellular ion gradients essential for cardiac function [8]. The crystal structure of Na^+^/K^+^-ATPase in complex with digoxin (PDB ID: 7DDH, 3.46 Å resolution) reveals the molecular basis of this interaction [9]. Digoxin binds to a preformed cavity within the extracellular domain of the α-subunit, positioned between transmembrane helices (TM) M1, M2, M4, M5, and M6 (Figure 1). The binding mechanism involves several critical molecular interactions (Figure 1). The steroid backbone of digoxin makes extensive hydrophobic contacts with the transmembrane region, while the sugar moieties extend toward the extracellular surface. A key hydrogen bond is formed between the hydroxyl group at position C14 of digoxin and Thr797, that are common interaction between Na^+^/K^+^-ATPase and cardiotonic steroids (CTS). Digoxin also has a van der Waals interaction between its C12-hydroxyl group and Gly319. Additionally, recent MD simulations have further clarified lipid-protein interactions in Na^+^/K^+^-ATPase. Lipid interactions change depending on the enzyme’s conformational state, and disease mutations can affect these interactions, providing new insights into digoxin binding. This research complements the structural findings, suggesting that lipid-protein interactions may play a role in stabilizing digoxin’s binding to the enzyme [9,10].

A comparative structural analysis based on the study by Kanai et al. (2021) provides key insights into digoxin’s mechanism of action [9]. In this study, the E2P ground state of the enzyme is represented by the E2·BeF_3_^-^ complex, free of CTS, and this state already possesses a preformed cavity. Digoxin binds to this preformed cavity, stabilizing the enzyme in the E2P form. Rather than restricting the enzyme’s flexibility, digoxin acts like a ‘doorstop’ to block the essential conformational transitions of the catalytic cycle, effectively ‘locking’ the enzyme. This is also evident in comparison with ouabain. While the steroid cores of both drugs occupy nearly identical positions, ouabain forms additional hydrogen bonds via hydroxyl groups on its β-face. In contrast, digoxin exhibits the unique feature of its C12-hydroxyl group interacting with the Gly319 residue of the M4 helix.

In the K^+^-bound state (represented by E2·MgF_4_^2-^·2K^+^), the M4 helix moves to close the extracellular domain more compactly, and digoxin physically obstructs this essential gating movement. Furthermore, this study uses the BeF_3_^-^ complex as an analog of the phosphorylated E2P state (the phosphorylated conformation of Na^+^/K^+^-ATPase), clearly demonstrating that digoxin acts as a potent inhibitor not by inducing the E2P state, but by selecting and stabilizing the pre-existing conformation.

The structural data demonstrates that digoxin’s therapeutic effect does not stem from competing with natural substrates, but from conformational trapping that disrupts the enzyme’s normal catalytic cycle [9,11,12]. Importantly, the structural data demonstrate that digoxin binding does not require large conformational changes in the Na^+^/K^+^-ATPase, contradicting earlier induced-fit models [13]. Instead, the drug occupies a binding site that is optimally shaped to accommodate the cardiac glycoside structure. This binding effectively locks the enzyme in the E2P state, preventing the conformational changes necessary for ion transport and resulting in increased intracellular Na^+^ levels [14]. This change ultimately enhances cardiac contractility through secondary effects on calcium handling [15,16,17].

### 2.2. Simvastatin and HMG-CoA Reductase: Inhibition of Cholesterol Biosynthesis

Simvastatin, introduced in 1988 as a semi-synthetic statin, inhibits 3-hydroxy-3-methylglutaryl-coenzyme A (HMG-CoA) reductase, the rate-limiting enzyme of cholesterol biosynthesis [18]. Simvastatin is administered as a therapeutically inactive lactone form and must undergo enzymatic hydrolysis in the body to be converted to its active metabolite, simvastatin β-hydroxy acid [19]. This bioactivation is primarily mediated by non-specific carboxylesterases and occurs in the intestinal wall, plasma, and liver. This conversion process transforms the highly lipophilic lactone (water solubility 0.0013~0.0015 mg/mL, approximately) into the more hydrophilic β-hydroxy acid. By contrast, the active metabolite in its ammonium salt form has a water solubility (~2 mg/mL) which is more than 1000-fold higher than the parent compound [20].

**Figure 2:**
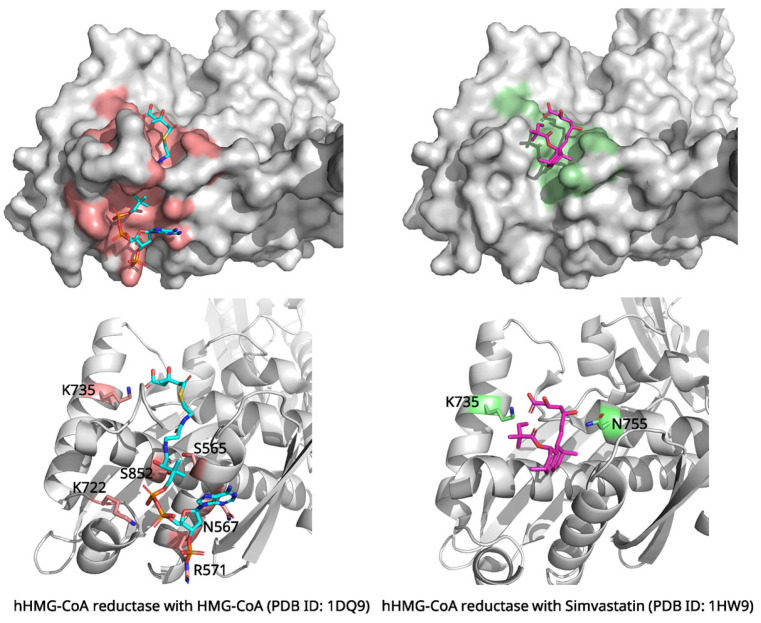
Comparison of human HMG-CoA reductase structure complex with HMG-CoA (**left**) and Simvastatin (**right**). HMG-CoA and simvastatin are colored cyan and magenta. C-terminus of hHMG-CoA reductase with simvastatin is not visible due to C-terminus rearrangement by binding with simvastatin. However, C-terminus of hHMG-CoA reductase is involved to bind with HMG-CoA. Key residues interacting with each ligand within the preformed binding cavities are highlighted and labeled in the enlarged views. Figures were created by PyMOL program with PDB ID: 1DQ9, 1HW9.

The crystal structure of human HMG-CoA reductase in complex with simvastatin (PDB ID: 1HW9, 2.3 Å resolution) provides insights into statin inhibition mechanisms [21]. The enzyme’s catalytic domain consists of a large domain containing the HMG-binding site and a small domain harboring the CoA-binding region (Figure 2). Statin β-hydroxy acids bind competitively in the HMG-binding pocket, mimicking the natural substrate while forming more favorable interactions.

Structural comparison between the substrate-bound (PDB ID: 1DQ9) and simvastatin-bound structures reveals the molecular basis of competitive inhibition (Figure 2) [22]. In the substrate-free state, the enzyme displays a disordered C-terminal region (residues N870-R871), creating an “open” active site. Upon HMG-CoA binding, this region becomes ordered, positioning catalytic His866 for activity through interactions with key residues including Lys735, Ser684, Asp690, and Lys691 [23]. Simvastatin achieves competitive inhibition through molecular mimicry—its β-hydroxy acid moiety perfectly overlays with the HMG portion of the natural substrate. This forms identical interactions including ionic bonding with Lys735 and hydrogen bonds with Ser684/Asp690 [21]. Recent QSAR (Quantitative Structure-Activity Relationship) modeling studies have provided insights into predicting the inhibitory potential of HMG-CoA reductase inhibitors, emphasizing the role of structural similarities in enhancing statin efficacy [24]. Additionally, simvastatin’s decalin ring system engages hydrophobic residues (Leu562, Val683, Leu853, Ala856, and Leu857) in a shallow groove formed by C-terminal rearrangement. Structural overlays demonstrate that simvastatin occupies the exact HMG-binding pocket. This directly blocks substrate access, which is the hallmark of competitive inhibition. The inhibitor prevents catalysis by occupying the active site while inducing conformational changes that eliminate catalytic competence, explaining the mechanism underlying statin efficacy [21].

Structural analysis reveals that the core pharmacophore of statins, the hydroxy acid moiety, is structurally analogous to the HMG portion of the natural substrate, HMG-CoA, thereby occupying the same binding site [21]. This hydroxy acid moiety forms several critical polar interactions within the enzyme’s active site, including an ionic bond with Lys735 and hydrogen bonds with residues such as Ser684, Asp690, and Asp767. Concurrently, hydrophobic groups, such as the decalin ring of simvastatin, bind to a separate hydrophobic groove. This groove is exposed through a flexible conformational change in the enzyme, providing additional binding affinity. This hydrophobic interaction involves extensive van der Waals contacts with residues such as Leu562, Val683, Leu853, Ala856, and Leu857. Thus, due to this additional hydrophobic binding, which is absent in the natural substrate, statins achieve a significantly higher binding affinity than the substrate. This effectively blocks the active site and exerts a potent competitive inhibitory effect [23].

A particularly important structural feature is the displacement of the mobile flap domain upon statin binding. This conformational change exposes additional hydrophobic surface area that accommodates the statin’s side chain, explaining the enhanced binding affinity compared to the natural substrate [21]. The structural data explain why statins are such effective inhibitors, with lovastatin showing a Ki of 0.6 nM compared to the KM of approximately 4 μM for HMG-CoA, representing an approximately 6700-fold higher binding affinity [25,26,27].

### 2.3. Morphine and μ-Opioid Receptor: Mechanisms of Pain Relief and Receptor Activation

Morphine, first isolated in 1804 from the opium poppy, marked the beginning of modern pharmacology and remains the reference opioid for severe pain management due to its well-characterized pharmacology and extensive clinical use [21,22,23].

**Figure 3:**
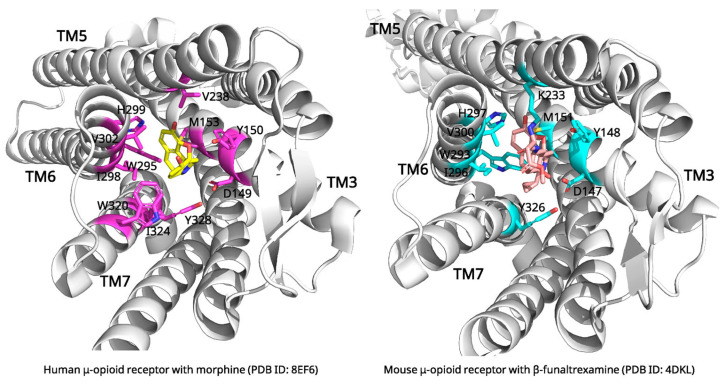
Complex structure of morphine-bound μ-opioid receptor (**left**) and β-funaltrexamine-bound μ-opioid receptor (**right**). Key residues that involve interacting with morphine (yellow) and β-funaltrexamine (salmon) labeled black. Figures were created by PyMOL program with PDB ID: 8EF6, 4DKL.

Although cryo-EM has a resolution limits (typically in the range of 2–4 Å that may restrict the detailed interpretation [28], recent advances in cryo-EM have provided unprecedented insights into opioid signaling mechanisms through high-resolution structures of the μ-opioid receptor (MOR). The cryo-EM structure of the morphine-bound MOR (PDB ID: 8EF6, 3.2 Å resolution) reveals the molecular basis of morphine binding [29]. Morphine binds in a large, solvent-accessible pocket formed by TM3, TM5, TM6, and TM7. The binding site accommodates the rigid morphine scaffold through a network of specific interactions (Figure 3).

Critical binding interactions include an ionic bond between morphine’s protonated amine and Asp149 in TM3, along with interactions with other key residues such as Tyr150 and His299 in TM6 [29]. The phenolic hydroxyl group of morphine points toward TM5, while other interactions, such as those involving the C6 hydroxyl group, are stabilized by a network of water molecules that bridge to receptor residues [30]. These interactions collectively stabilize morphine in an orientation that promotes receptor activation [29].

Comparative structural analysis reveals distinct conformational states that explain the pharmacological mechanisms of different opioid ligands (Figure 3). Recent studies combine MD simulations and ML approaches to analyze the biased activation mechanisms of the μ-opioid receptor, shedding light on how specific residues contribute to biased signaling states [31]. Inactive receptor structures, such as the β-funaltrexamine-bound complex (PDB ID: 4DKL), display characteristic features including an inward-positioned TM6 and an occluded G-protein binding cavity [32]. This inactive state is further stabilized by a key polar interaction between Arg165 on TM3 and Thr279 on TM6, which functions as an “ionic lock (a stabilizing polar interaction that keeps the receptor in an inactive conformation)” [30,33]. Antagonists function by stabilizing these inactive conformations, thereby preventing the activation cascade required for signaling [32]. In contrast, endogenous peptide-bound structures reveal a more extended receptor-ligand interface compared to the compact footprint of small molecules like morphine [34]. Natural opioid peptides primarily engage the orthosteric binding pocket through their conserved N-terminal tyrosine residue. This residue serves as the key “message” for receptor activation and creates peptide-specific activation dynamics [35,36].

Morphine’s dual binding mode, engaging in both TM3 and TM6/7 regions, distinguishes it from G-protein-biased agonists [29]. Key activation features include the rotation of the Trp295 toggle switch (an aromatic side chain whose rotation is linked to receptor activation), the breaking of an ionic lock between Arg165 on TM3 and Thr279 on TM6, and a characteristic outward displacement of TM6 (approx. 10 Å) that creates the G-protein binding cavity [30,32]. Unlike G-protein-biased agonists, morphine and other balanced agonists (including endogenous peptide analogues) make extensive contacts with the TM6/7 region, which enables the recruitment of both G-protein and β-arrestin pathways [29]. This dual activation mechanism explains morphine’s therapeutic efficacy alongside its side effects, including tolerance and respiratory depression. It also provides structural insights for developing biased opioid agonists with improved therapeutic profiles.

The structural data reveal important insights into functional selectivity, a phenomenon where different ligands can bias receptor signaling toward different downstream pathways. Comparative analysis between structures bound to balanced agonists (like morphine or DAMGO (([D-Ala^2^, *N*-MePhe^4^, Gly-ol]-enkephalin)) and those bound to G-protein-biased agonists shows specific conformational changes that favor G-protein coupling over β-arrestin recruitment [29]. This structural understanding has provided a foundation for recent developments in biased opioid agonists. These agonists are designed to minimize side effects while maintaining analgesic efficacy.

### 2.4. Paclitaxel and β-Tubulin: Mechanisms of Microtubule Stabilization

Paclitaxel (Taxol), discovered in 1971 from the Pacific yew *Taxus brevifolia*, overcame initial supply and formulation challenges through semisynthetic production and was approved by the FDA in the early 1990s, revolutionizing cancer chemotherapy as one of the most important anticancer agents [1,31,32,33].

**Figure 4:**
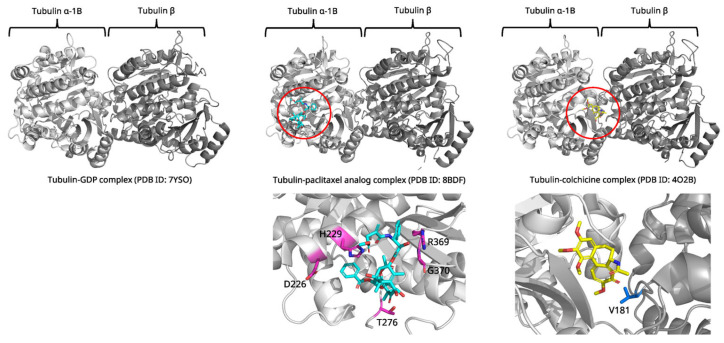
Structural comparison of tubulin complex with GDP (**left**), paclitaxel analog (**middle**) and colchicine (**right**). Paclitaxel and colchicine are colored cyan and yellow in each red circle. Paclitaxel and colchicine bind to different places in tubulin complex. Key residues interacting with each ligand are highlighted and labeled in the enlarged views. Figures were created by PyMOL program with PDB ID: 7YSO, 8BDF, 4O2B.

Unlike most antimitotic agents that destabilize microtubules, paclitaxel promotes microtubule polymerization and prevents depolymerization, effectively freezing the mitotic spindle [37]. High-resolution structures of tubulin in complex with paclitaxel have been solved using X-ray crystallography approaches (PDB ID: 8BDF, 1.9 Å resolution, Figure 4) [38]. Paclitaxel binds to a pocket on the inner surface of β-tubulin, facing the hollow interior of the microtubule. This binding site, known as the taxane site (the luminal pocket in β-tubulin where paclitaxel binds to stabilize microtubules), is formed by the M-loop (a flexible β-tubulin segment critical for lateral protofilament contacts, residues 274–286), helix H7, and surrounding β-sheet structures.

The binding mechanism involves several key structural elements. The baccatin III core of paclitaxel makes extensive hydrophobic contacts with the M-loop and helix H7. The C13 side chain containing the N-benzoyl-β-phenylisoserine moiety extends into a hydrophobic cleft formed by adjacent tubulin molecules. Critical hydrogen bonds are formed between the C2’ hydroxyl of paclitaxel and His229, and between the C7 hydroxyl and Arg369 [38,39].

Recent MD studies have revealed that paclitaxel binding induces conformational changes in the M-loop that propagate throughout the tubulin polymer, stabilizing lateral contacts between protofilaments (a linear chain of tubulin dimers that laterally associate to form microtubules) [40,41]. This structural information explains how paclitaxel achieves its unique mechanism of microtubule stabilization. It has also informed the development of next generation taxane analogs with improved therapeutic properties [42,43].

Comparative structural analysis reveals the mechanistic basis for paclitaxel’s stabilizing effects versus destabilizing agents (Figure 4). Native tubulin exists in curved conformations (PDB ID: 7YSO) with disordered M-loops incompatible with lateral protofilament contacts [44]. Destabilizing agents exploit this curvature through distinct binding mechanisms. For example, colchicine (PDB ID: 1SA0, 4O2B) occupies the β-tubulin intradimer interface, stabilizing the T7 loop in a “flipped-out” conformation that prevents the curved-to-straight transition required for assembly [45,46]. Vinblastine (PDB ID: 1Z2B) inserts as an interdimer wedge, disrupting longitudinal contacts and promoting spiral aggregates rather than functional microtubules [47].

In stark contrast, paclitaxel (PDB ID: 1JFF, 8BDF) binds the luminal taxane site accessible only in assembled microtubules, exhibiting 10,000-fold higher affinity for polymerized versus free tubulin [38,48,49]. Upon binding, paclitaxel stabilizes the M-loop (β275–286) in an α-helical conformation essential for lateral contacts, reverses GDP-induced compaction, and induces a stable state even with GDP-bound tubulin [50]. The key structural distinction lies in conformational selection. Destabilizers trap curved, assembly incompetent states through intradimer or interdimer binding, while paclitaxel recognizes and stabilizes the straight, assembled conformation through M-loop stabilization and allosteric modulation. This explains their opposite pharmacological effects—destabilizers prevent assembly by conformational restriction, whereas paclitaxel prevents disassembly by enhancing lateral contacts and suppressing the structural transitions underlying dynamic instability [51].

### 2.5. Penicillin and Penicillin-Binding Proteins: Inhibition of Bacterial Cell Wall Synthesis

**Figure 5:**
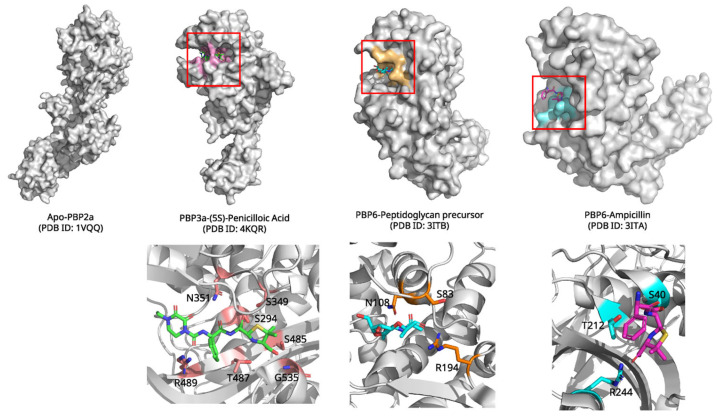
Structural comparison of PBPs with penicilloic acid, peptidoglycan precursor and ampicillin. Each binding molecule of PBPs is indicated by red box. Key residues interacting with each ligand are highlighted and labeled in the enlarged views. Figures were created by PyMOL with PDB ID: 1VQQ, 4KQR, 3ITB, 3ITA.

Penicillin, first discovered by Alexander Fleming in 1928 and developed into a therapeutic by Florey and Chain during World War II, revolutionized medicine as the first effective antibiotic, inaugurating the antibiotic era and saving millions of lives worldwide [46,47,48,49,50]. This β-lactam antibiotic targets penicillin-binding proteins (PBPs), essential enzymes involved in bacterial cell wall biosynthesis [52]. The crystal structure of penicillin in complex with PBP3 from *Pseudomonas aeruginosa* (PDB ID: 4KQR, 2.1 Å resolution, Figure 5) reveals the molecular mechanism of β-lactam action [53]. PBPs are serine proteases that catalyze the cross-linking of peptidoglycan strands in bacterial cell walls. Penicillin acts as a mechanism-based inhibitor, forming a covalent adduct with the catalytic serine residue.

The inhibition mechanism proceeds through nucleophilic attack by Ser294 on the β-lactam carbonyl carbon, resulting in ring opening and formation of a stable acyl-enzyme intermediate. The penicilloyl moiety becomes covalently attached to the enzyme, effectively blocking the active site and preventing peptidoglycan cross-linking [54]. Unlike normal acyl-enzyme intermediates formed during catalysis, the penicilloyl-enzyme complex is remarkably stable due to conformational strain and electrostatic interactions that inhibit hydrolysis [55].

Comparative structural analysis reveals the molecular basis of PBP inhibition through examination of different binding states (Figure 5). Apo PBP structures, such as *S. aureus* PBP2a (PDB ID: 1VQQ), display a “closed” active site conformation with poorly positioned catalytic residues [56]. Natural substrate binding, exemplified by *E. coli* PBP6 complexed with peptidoglycan precursor (PDB ID: 3ITB), induces an “open” configuration that accommodates the D-Ala-D-Ala terminus through conserved SXXK, SXN, and KTG motifs [57]. β-lactam binding triggers distinct conformational changes compared to natural substrates. Penicillin complexes reveal covalent acyl-enzyme formation with half-lives of 10–20 h versus seconds for physiological substrates. Critical structural rearrangements include β3 strand twisting to optimize oxyanion hole geometry and β3-β4 loop movement varying by approximately 4 Å in *P. aeruginosa* PBP3 [54].

Comparison of different β-lactam complexes elucidates selectivity determinants. Ampicillin (PDB ID: 3ITA) shows enhanced PBP6 binding through additional benzyl side chain contacts. In contrast, methicillin’s bulky substituents reduce PBP2a acylation rates to 0.001–0.2 s^−1^ [56,57]. Carbapenems like imipenem achieve broad-spectrum activity through extensive hydrogen bonding networks involving their 6α-1R-hydroxyethyl groups [58]. These structural insights reveal how different β-lactams exploit common active site features. They achieve varying potency and selectivity profiles through distinct molecular recognition patterns.

Structural studies have also elucidated the molecular basis of β-lactam resistance, particularly through the evolution of β-lactamases that can hydrolyze the antibiotic before it reaches its target. Recent high-resolution structures of various PBP-penicillin complexes have informed the design of new β-lactam antibiotics capable of overcoming resistance mechanisms [59]. A recent study combines structural biology experiments and MD simulations to evaluate the binding modes of novel β-lactone-based PBP inhibitors, providing further insights into the mechanisms of inhibition and selectivity in these compounds [60].

## 3. Comparative Structural Analysis of Natural Products: Common Binding Mechanisms

The structural analysis of these natural product-protein complexes reveals fundamental principles that explain their efficacy as therapeutic agents. A central theme is the exceptional degree of complementarity these molecules achieve with their targets, which is accomplished through a sophisticated interplay of precise structural recognition and the modulation of protein conformation.

A primary characteristic is the remarkable binding site complementarity, where each natural product achieves high affinity by precisely matching the geometry and chemistry of its target site. This superior fit often surpasses that of the endogenous substrate, explaining their potency as biological modulators. Simvastatin, for instance, competitively inhibits HMG-CoA reductase by occupying the substrate binding site. Its HMG-like moiety binds to the active site, while its hydrophobic groups exploit the enzyme’s flexibility to create an additional binding pocket, leading to nanomolar inhibition constants [21]. Penicillin acts as a mechanism-based inhibitor, forming a stable, long-lived covalent acyl-enzyme complex with the catalytic serine of PBPs, which effectively inactivates the enzyme [52].

These compounds employ a multimodal strategy, utilizing a combination of hydrogen bonds, hydrophobic contacts, and electrostatic interactions to secure high affinity and specificity. For example, paclitaxel’s binding to β-tubulin involves extensive hydrophobic contacts from its rigid core, while its C-13 side chain forms a critical hydrogen bond via its 2′-hydroxyl group [48]. Similarly, digoxin’s steroid core nestles into a hydrophobic cavity in the Na^+^/K^+^-ATPase, stabilized by polar interactions from its hydroxyl groups and lactone ring [61]. Morphine’s interaction with the MOR also involves a complex network of interactions that stabilize the active conformation [32]. This use of multiple, synergistic interactions contributes to their robustness as therapeutic scaffolds.

Perhaps most sophisticated is how these natural products harness protein dynamics, employing mechanisms that range from induced fit to conformational selection. Digoxin exemplifies pure conformational selection; it does not induce a major structural change but instead binds to a pre-formed cavity present only in the transient E2P state of the Na+/K+-ATPase [62]. This binding traps the enzyme, preventing the conformational cycling required for ion transport. In contrast, morphine binding to the MOR triggers an induced-fit cascade, causing a significant outward movement of TM6 to create the G-protein binding site necessary for signal activation [63]. Paclitaxel employs a hybrid mechanism; it selectively recognizes the assembled microtubule structure, where the M-loop of β-tubulin is already in a favorable “out” conformation, but its binding then further stabilizes this state, allosterically reinforcing the lateral contacts between protofilaments and preventing disassembly [64].

Collectively, these examples demonstrate that natural products are exquisitely tuned modulators of protein function. Their ability to achieve superior binding site complementarity and utilize diverse chemical interactions explains their enduring value as therapeutic agents. Additionally, they masterfully exploit the conformational landscapes of their targets, positioning them as blueprints for future drug discovery [1].

Each mechanism presents distinct strengths and limitations: covalent β-lactam acylation provides durable inhibition but is vulnerable to resistance, statin competitive inhibition offers tunability yet requires precise active-site mimicry, and paclitaxel’s conformational selection ensures robust stabilization though it can vary across tubulin isotypes. Morphine and digoxin further illustrate how induced fit and conformational trapping confer efficacy but also impose context-dependent constraints. These contrasts underscore the diversity of strategies employed by natural products.

**Figure 6:**
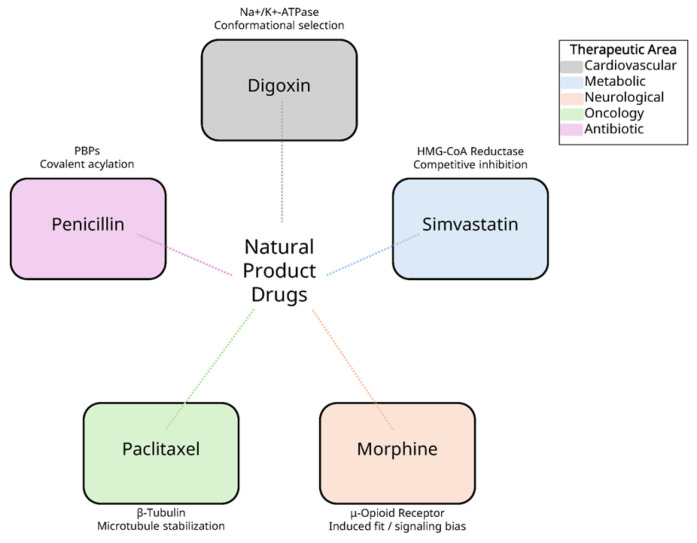
Integrative schematic of representative natural product-derived drugs, their primary protein targets, binding mechanisms, and therapeutic areas. This conceptual map highlights how digoxin, simvastatin, morphine, paclitaxel, and penicillin exemplify diverse mechanistic strategies—conformational selection, competitive inhibition, induced fit/signaling bias, microtubule stabilization, and covalent acylation—while spanning major therapeutic domains (cardiovascular, metabolic, neurological, oncological, and antibiotic).

To highlight these common principles and provide a concise comparison, Table 1 summarizes the representative natural product-derived drugs discussed in this review, along with their protein targets, binding mechanisms, and key structural insights. Complementing this tabular summary, Figure 6 presents an integrative schematic that conceptually maps these drugs to their binding modes and therapeutic areas, thereby providing an overarching view that reinforces the detailed information in Table 1.

## 4. Conclusions

The structural analyses presented in this review demonstrate that natural product-derived drugs continue to represent an invaluable foundation for modern therapeutics, with their molecular mechanisms now elucidated at unprecedented atomic resolution [65,66]. Through examination of five clinically important natural products—digoxin, simvastatin, morphine, paclitaxel, and penicillin—we have revealed the diverse strategies. These strategies explain how these compounds achieve their remarkable therapeutic effects.

A striking finding from our comparative analysis is the sophistication of molecular recognition exhibited by these natural products. Each compound demonstrates exquisite complementarity to its protein target, achieved through millions of years of evolutionary optimization. This complementarity extends beyond simple shape matching to encompass precise electrostatic, hydrophobic, and hydrogen bonding networks that collectively confer both high affinity and specificity. The structural data reveal that natural products often surpass synthetic compounds in their ability to exploit multiple binding subsites simultaneously. This is exemplified by simvastatin’s dual occupation of both the substrate-binding pocket and an induced hydrophobic groove in HMG-CoA reductase [67].

Perhaps most significantly, these structural studies have illuminated the diverse mechanisms through which natural products modulate protein function. We observe a spectrum of strategies ranging from competitive inhibition (simvastatin), covalent modification (penicillin), conformational selection (digoxin), induced-fit activation (morphine), to allosteric stabilization (paclitaxel). This mechanistic diversity underscores the versatility of natural product scaffolds and their ability to target proteins through multiple modalities that would be challenging to design de novo.

The availability of high-resolution structures has profound implications for future drug development. These structures serve as molecular blueprints for rational drug design, enabling the optimization of natural product scaffolds to enhance therapeutic properties while minimizing adverse effects [6]. By linking atomic-level mechanisms with clinical translation—for example, the design of biased opioid agonists for safer analgesia or the optimization of β-lactams to overcome resistance—structural biology directly informs the development of next-generation therapeutics. Detailed understanding of binding modes facilitates the design of biased ligands, as demonstrated by recent developments in opioid agonists that selectively activate beneficial signaling pathways [68]. Furthermore, structural insights into resistance mechanisms, particularly evident in β-lactam antibiotics, guide the development of next-generation compounds capable of overcoming evolved resistance [69].

Looking forward, several emerging trends warrant attention. The application of cryo-EM is revolutionizing our understanding of natural product interactions with membrane proteins and large macromolecular complexes, previously intractable to crystallographic analysis [70]. Advanced computational methods, including MD simulations and ML approaches, are increasingly complementing experimental structures to provide dynamic insights into natural product-protein interactions [71]. Additionally, the integration of structural biology with synthetic biology approaches promises to unlock new natural product diversity through engineered biosynthetic pathways [72]. Emerging single-cell and spatial omics, together with advanced imaging such as time-resolved cryo-EM and single-molecule FRET (smFRET) (exemplified by recent applications resolving protein dynamics under time-resolved protocols [73,74]), will contextualize ligand effects across cellular states and capture transient intermediates beyond static snapshots. Recent therapeutic advances illustrate how structural insights are being translated into clinical applications. For example, proof-of-concept studies demonstrated the potential of digoxin in disrupting circulating tumor cell clusters [75], while updated safety and efficacy evaluations of statins [76,77] inform their ongoing clinical use. Similarly, recent work on opioid receptor bias [78,79,80], paclitaxel isoform-specific binding [38,42,81], and novel β-lactam strategies against resistant pathogens [82,83,84] underscores the translational relevance of these mechanistic findings.

Despite these advances, significant challenges remain. Many natural product targets, particularly those involving transient protein–protein interactions or intrinsically disordered proteins, remain structurally uncharacterized [85]. The complexity of natural product biosynthesis continues to limit supply for many promising compounds, though structural understanding of biosynthetic enzymes offers pathways to biotechnological solutions [86]. Furthermore, the development of resistance, particularly prominent in antimicrobial and anticancer applications, necessitates continuous structural investigation to maintain therapeutic efficacy.

Beyond resistance and supply, clinical use of natural products is constrained by narrow therapeutic indices, as seen with cardiotonic steroids, by off-target liabilities, as observed with statins, and by tolerance development, as exemplified by opioids, underscoring the need for mechanism-informed risk mitigation. Translational barriers include scalable supply for complex scaffolds, chemistry–manufacturing–control (CMC) constraints for semisynthetic routes, intellectual property (IP) considerations for natural product derivatives, and Investigational New Drug (IND)-enabling toxicity studies aligned with the mechanism. Key gaps remain, including structural characterization of intrinsically disordered drug targets, lack of pan-isotype tubulin structures, and limited in vivo validation of biased GPCR signaling.

In conclusion, natural products remain an essential source of therapeutic agents, with structural biology providing the molecular foundation for understanding and optimizing their biological activities. The five examples presented here—spanning cardiovascular, metabolic, neurological, oncological, and antimicrobial applications—demonstrate that natural products achieve their effects through sophisticated molecular mechanisms that continue to inspire drug discovery. As structural biology techniques advance and our understanding of natural product-protein interactions deepens, these ancient remedies will undoubtedly continue to yield modern medicines. By unifying static structures with dynamic insights across archetypal natural products, this review provides a mechanistic framework that directly informs analog design, signaling bias engineering, and resistance-aware optimization. These integrative perspectives highlight the unique contribution of this review in bridging fundamental structural principles with translational applications, offering a blueprint for guiding future natural product–based drug discovery.

## Figures and Tables

**Table 1:** Comparative summary of representative natural product-derived drugs and related compounds, their protein targets, binding mechanisms, and key structural insights.

Drug	Protein Target	Binding Mechanism	PDB ID (Resolution, Method)	Structural Insights
Digoxin	Na^+^/K^+^-ATPase	Conformational selection; stabilizes E2P state, blocks catalytic cycle transitions	7DDH (3.46 Å, X-ray)	Binds preformed cavity (M1, M2, M4-M6); hydrophobic contacts and H-bonds (Thr797, Gly319)
Simvastatin	HMG-CoA Reductase	Competitive inhibition; substrate mimicry	1HW9 (2.30 Å, X-ray)	Hydroxy acid moiety mimics HMG; H-bonds with Lys735, Ser684, Asp690; hydrophobic decalin ring interactions (Leu562, Val683, etc.)
Morphine	μ-Opioid Receptor	Induced fit; receptor activation via TM6 displacement	8EF6 (3.20 Å, Cryo-EM)	Ionic bond with Asp149; H-bonds with Tyr150, His299; stabilizes active conformation enabling G-protein/p-arrestin recruitment
Paclitaxel	β-Tubulin	Conformational selection; stabilizes microtubule assembly	8BDF (1.90 Å, X-ray)	Binds taxane site (M-loop, H7); H-bonds with His229, Arg369; stabilizes lateral protofilament contacts
Penicillin	Penicillin-Binding Proteins	Covalent inhibition; acyl-enzyme intermediate formation	4KQR (2.10 Å, X-ray)	Forms covalent bond with Ser294; blocks peptidoglycan cross-linking; P3–P4 loop rearrangements explain inhibition/selectivity

## Data Availability

No new data were created or analyzed in this study.
